# Examining the role of personality functioning in a hierarchical taxonomy of psychopathology using two years of ambulatory assessed data

**DOI:** 10.1038/s41398-024-03046-z

**Published:** 2024-08-24

**Authors:** André Kerber, Johannes C. Ehrenthal, Johannes Zimmermann, Carina Remmers, Tobias Nolte, Leon P. Wendt, Phileas Heim, Sascha Müller, Ina Beintner, Christine Knaevelsrud

**Affiliations:** 1 Division of Clinical-Psychological Intervention, Freie Universität Berlin, Berlin, Germany; 2 Department of Psychology, University of Cologne, Cologne, Germany; 3 Department of Psychology, University of Kassel, Kassel, Germany; 4 Department of Psychology, Institute for Mental Health and Behavioral Medicine, HMU Health and Medical University, Potsdam, Germany; 5 Research Department of Clinical, Educational and Health Psychology, UCL, and Anna Freud, London, UK; 6 Department of Psychosomatics and Psychotherapy, University Medical Center Rostock, Rostock, Germany; 7 MindDoc Health GmbH, Munich, Germany

**Keywords:** Psychiatric disorders, Pathogenesis

## Abstract

The Hierarchical Taxonomy of Psychopathology (HiTOP) arranges phenotypes of mental disorders based on empirical covariation, ranging from narrowly defined symptoms to higher-order spectra of psychopathology. Since the introduction of personality functioning (PF) in DSM-5 and ICD-11, several studies have identified PF as a predictor of transdiagnostic aspects of psychopathology. However, the role of PF in the HiTOP classification system has not been systematically examined. This study investigates how PF can be integrated into HiTOP, whether PF accounts for transdiagnostic variance captured in higher-order spectra, and how its predictive value for future affective well-being (AWB) and psychosocial impairment (PSI) compares to the predictive value of specific psychopathology beyond PF. To this end, we examined two years of ambulatory assessed data on psychopathology, PF, PSI, and AWB of *N* = 27,173 users of a mental health app. Results of bass-ackwards analyses largely aligned with the current HiTOP working model. Using bifactor modeling, aspects of PF were identified to capture most of the internalizing, thought disorder, and externalizing higher-order factor variance. In longitudinal prediction analyses employing bifactor-(S-1) modeling, PF explained 58.6% and 30.6% of variance in PSI and AWB when assessed across one year, respectively, and 33.1% and 23.2% of variance when assessed across two years. Results indicate that personality functioning may largely account for transdiagnostic variance captured in the higher-order components in HiTOP as well as longitudinal outcomes of PSI and AWB. Clinicians and their patients may benefit from assessing PF aspects such as identity problems or internal relationship models in a broad range of mental disorders. Further, incorporating measures of PF may advance research in biological psychiatry by providing empirically sound phenotypes.

## Introduction

### Hierarchical taxonomy of psychopathology

Decades of research on psychopathology indicate that categorical approaches to assessment are limited [1, 2]. Emerging models, such as the Hierarchical Taxonomy of Psychopathology (HiTOP [3]), adopt a dimensional perspective, prioritizing empirical data over expert consensus [4]. Following this approach, comorbidity is not a validity problem but an inherent and empirically supported aspect of the classification system. Consequently, mental health problems that tend to co-occur in individuals can aid in finding more general, higher-order factors of psychopathology that show distinct genetic, neurobiological, environmental, and behavioral correlates [3, 5–7]. These empirically derived phenotypes have the potential to advance psychiatric genetics [8] and to provide clinical utility in everyday practice [9]. Current reviews on the HiTOP model propose a superspectrum of emotional dysfunction, which includes somatoform and internalizing spectra. These, in turn, encompass subfactors such as fear, distress, eating pathology, or sexual problems, which further narrow down to individual symptoms (e.g., dysphoria) and traits (e.g., separation insecurity). The superspectrum of psychosis includes spectra of thought disorder and detachment, while the superspectrum of externalizing encompasses disinhibited and antagonistic spectra, including the subfactors harmful substance use and antisocial behavior. At the highest level of the hierarchy, the “*p*-factor” represents the empirical covariance between all mental disorders. In summary, HiTOP provides a comprehensive taxonomy that enables a multidimensional, hierarchical classification of mental health problems supported by meta-analytic evidence [10].

Notably, HiTOP also includes maladaptive personality traits based on the assumption that the difference between symptoms and traits primarily lies in the timeframe of occurrence [11]. It hereby incorporates findings from personality psychology regarding the convergence of HiTOP spectra and maladaptive trait domains [12]. Additionally, including dimensionally assessed traits aligns with research showing that maladaptive personality traits predict the onset of psychopathology, symptom chronicity, and functioning above and beyond categorical diagnoses [13].

### Personality functioning (PF) and HiTOP

Both DSM-5, section III [14], and the new ICD-11 model of personality disorders [15–17] place the dimensional assessment of impairments in self- and interpersonal functioning (i.e., personality functioning) at the center of their approach. Unlike personality traits, which describe *how* individuals are, personality functioning (PF) focuses on basic psychological capacities individuals possess in perception, regulation, communication, and relationship formation to interact with themselves and the social world [18]. This definition of PF draws on objects relations and mentalization theories [14], which postulate that deficits in regulating the self and relationships (i.e., low levels of PF) are a result of adverse gene-environment interactions in early childhood and predispose individuals to psychopathology in general [19]. Empirically, PF shows less longitudinal stability in mean levels compared to personality traits, except for neuroticism [20], which overlaps significantly with PF [21] and appears more responsive to clinical interventions than other traits [22]. Additionally, PF is associated with various variables related to personality disorders, psychopathology, and psychosocial functioning [23–25]. Longitudinal research demonstrates that impairment in PF is a stronger predictor of psychosocial functioning than the sum of DSM-IV personality disorder criteria [26] or maladaptive traits [27].

Widiger et al. [28] proposed to integrate PF in the HiTOP system by mapping it largely on the *p*-factor of the model, which was supported by Bender [29]. Meehan et al. [30] argued that incorporating PF into HiTOP could help to “more fully capture the complexity of personality pathology over time” (p. 372). They also suggested that PF reflects the unstable and dynamic aspects of PDs while maladaptive traits may account for the stability of specific (PD) phenotypes, a hypothesis that has accumulated empirical evidence lately [31, 32]. In a recent study investigating a large battery of established questionnaires regarding their alignment with HiTOP using a cross-sectional sample, some PF scales mapped closely onto distinct spectra, whereas other PF scales including mentalizing, negative interpersonal relationships, and problems with emotion awareness aligned with blends of spectra or lacked specificity for any particular spectrum, suggesting they are “pure markers” of the *p*-factor [33]. However, studies with sufficient statistical power, ecological validity, or longitudinal data on the role of PF in HiTOP are lacking.

### Current study

In this study, we investigated three research questions: (1) How does PF fit into a hierarchical taxonomy of psychopathological symptoms and maladaptive traits? Does it represent a homogeneous construct that can be allocated to a specific subfactor or spectrum? (2) Does PF account for transdiagnostic variance captured in the higher-order factors? (3) If so, how much predictive validity does PF have, and how much predictive validity does specific psychopathology have beyond PF? To this aim, first, we utilized the extended bass-ackwards procedure [34] on ambulatory assessed psychopathological symptoms, traits, and PF of 27,173 users of a mental health app. We examined the placement of PF within an empirically derived hierarchical structure and explored whether PF accounted for general or specific variance in HiTOP using bifactor models. Additionally, we investigated the predictive validity of PF and residualized specific factors in relation to future affective well-being and psychosocial impairment using bifactor-(S-1) models.

## Methods

### Data collection

**Fig. 1:**
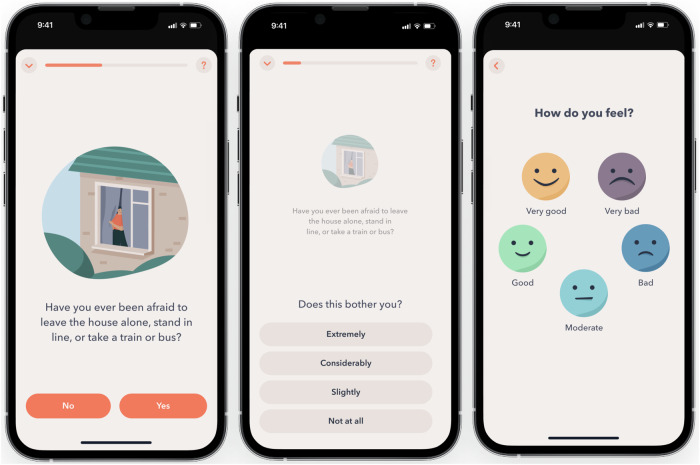
Assessment system in the MindDoc app.

We analyzed anonymized data from the MindDoc app, which is a self-guided transdiagnostic application for individuals seeking to manage their mental health. It can be used anonymously, and according to the GDPR principle of data minimization, no sociodemographic data is collected. However, in a separate questionnaire study of *N* = 1010 MindDoc users, 93.9% showed symptoms of depression and/or anxiety, 65.4% had outpatient and 49.2% had inpatient treatment in the last 6 months (see https://osf.io/swj3c/ for more details). A detailed description of all features of the app as well as its effectiveness can be found elsewhere [35–37]. The app is available on the Appstore and Playstore in German and English as a commercial product with both free and paid features. In addition to courses and exercises, it offers self-monitoring of psychopathology, psychosocial functioning, and personal resources. The self-monitoring feature is fully usable without a paid subscription and consists of three daily assessment blocks with three to nine questions aligned with the user’s circadian rhythm. Depending on previous answers, psychopathology, psychosocial impairment, and resources are adaptively explored using questions. Questions on psychopathology are regularly and repeatedly interspersed between all other questions in the ambulatory assessment, and the algorithm ensures that all HiTOP spectra are explored, leading to multiple assessments of the same question in regular users. Questions are first asked in a dichotomous yes/no format followed by a four-point scale assessing intensity or how much a statement applies, depending on item content, yielding a 5-point scale. Users also rate their current affective well-being after each question block (see Fig. 1).

From a database of *N* = 157,212 users, we included *N* = 27,143 active users with at least one assessment of at least 90% of 201 ambulatory assessed items capturing psychopathology and psychosocial impairment. Psychopathology assessments were available for a 10-month period (2021-06-01 to 2022-04-01), while assessments of affective well-being and psychosocial impairment were available for a 24-month period (2021-06-01 to 2023-06-01). Note that for the prediction of affective well-being (*N* = 25,844 over one year; *N* = 10,636 over two years) and psychosocial impairment (*N* = 27,173 over one year; *N* = 5342 over two years), sample sizes were somewhat smaller because for these analyses, we only included participants with at least two assessment within these time periods. The number of assessments varied between individuals depending on app usage duration and individual psychopathology. Mean and range of assessment frequency as well as the distribution of the 98 psychopathology scales and user attrition for the included users within the two-year period of assessment are available in the Supplemental material.

The feasibility and validity of this assessment method for psychopathology was investigated in a previous version of the app [38]. The procedure of data transfer and processing was approved by a local ethics committee of FU Berlin, Department of Psychology (Nr. 047/2020). Informed consent was obtained from all subjects that provided personal data (Ethics vote Nr. 038/2022). All data collection and analysis methods were performed in accordance with the Helsinki Declaration on Medical Research Involving Human Subjects as well as the European General Data Protection Regulation.

### Measures

#### Assessment of psychopathology

Psychopathology items were developed by a board of licensed clinical psychologists, psychiatrists, and clinical psychology researchers to capture diagnostic criteria (symptoms) for mental disorders (team including AK and IB), maladaptive traits (AK, JZ, and LW), and personality functioning (AK, JCE, and TN). Table 1 lists all psychopathology scales used in this study, including example items. Questions on symptoms of mental disorders (71 scales, 128 items) were aligned with diagnostic criteria from ICD-11. Some disorders had general opening questions that triggered further questions. For example, for eating disorders (ED), affirming the opening question “Have you set up certain rules, prohibitions, or patterns about eating?” prompts subsequent questions on eating behavior. If the opening question was negated, all subsequent questions related to that disorder were assigned a value of 0 for further analyses. PF was assessed with 11 scales (27 items) that were identified as highly indicative of the severity of personality dysfunction based on previous data from the Operationalized Psychodynamic Diagnosis - Structure Questionnaire (OPD-SQ [18, 39]) in clinical samples [40]. The original OPD-SQ [39, 41] and its short form (OPD-SQS [42, 43]) are self-reports for measuring impairments in PF that are consistently associated with interview-based measures of PF according to the DSM-5 AMPD [44, 45] and other related constructs [46–48]. Items were reformulated to match the format of questions in the app (e.g., from “I sometimes feel like a stranger to myself” of the original OPD-SQ to “Do you sometimes feel like a stranger to yourself?”). Questions on maladaptive traits (22 scales, 5 domains, 40 items) were aligned with Criterion B of the DSM-5 AMPD. However, a low number of items and avoidance of redundancy in item content was a prerequisite for integrating the assessment in the App. Besides leaving out interstitial trait facets submissiveness and attention seeking, this led to omission of negative affectivity facets emotional lability and anxiety due to high redundancy with PF facet affect tolerance and items for generalized anxiety disorder, respectively as well as disinhibition facet distractibility and detachment facet depressivity due to redundancy with items for depression.

**Table 1:** Ambulatory assessment of psychopathology.

Type	Domains and subfacets	Example items
**Symptoms**	**Depression (DEP):** depressed mood [2], diminished interest in activities [3], hopelessness [3], reduced energy [3], circadian mood fluctuations [1], concentration difficulties [2], crying [1], crying inability [1], decision problems [1], feeling of emotional numbness [1], feelings of guilt [1], hypersomnia [1], increased appetite [1], loss of sexual libido [1], loss of appetite [1], diminished self confidence [2], psychomotric inhibition [1], psychomotoric agitation [1], rumination [1], feelings of worthlessness [1], suicidal intentions [1]	“Are you feeling down and sad?” (DEP_depr_mood); “Do you have less pleasure in doing things you usually enjoy?” (DEP_dimin_interest); “Do you think you are worth less than others right now?” (DEP_dimin_selfconf); “Are you speaking or moving more slowly than usual?” (DEP_psycmot_inhib) “Are you feeling hopeless?” (DEP_hopelessn)
	**Manic symptoms** [3]	“Have you recently been sleeping considerably less than usual, yet have an unusually high level of energy nonetheless?” (Manic_sympt)
	**Agoraphobia** [7]	“Are you afraid of being in a situation you can’t leave or where you think you may be unable to get help if needed?” (Agoraphobia)
	**Generalized anxiety disorder (GAD):** subjective experience of nervousness [2], free floating anxiety [4], excessive worrying [5], irritability [1]	“Are you often tired and exhausted because of your worries around a variety of issues?” (GAD_free_float_anx)
	**Social anxiety disorder (SAD):** social interactions [2], doing tasks while being observed [1], performing in front of others [4], eating in front of others [4], fear of devaluation [2], avoidance of interactions with strangers [2], interacting with attractive persons [1]	“Do you feel extremely uncomfortable in social situations, or are you afraid to speak, eat, or write in front of others?” (SAD_soc_inter) Do you feel uncomfortable or even frightened at the very thought of doing mundane tasks like writing in front of others or paying at the register? (SAD_being_observ)
	**Specific phobia:** excessive fear of certain objects [3], animals [1], blood-injury [1], avoidance of feared objects [1]	“Are there any particular objects or situations you are intensely afraid of, such as the sight of blood or a certain insect or animal, or flying in a plane?” (Spec_phob_fear)
	**Panic disorder:** panic attacks [2], fear of recurring panic attacks [1], avoidance behavior [1], physical symptoms [1], doctor visits due to panic attacks [1], dysfunctional thoughts during panic attacks [3]	“Have you had a sudden panic attack within the last few weeks?” (Panic_attacks) “Have you thought you might be dying when having a panic attack?” (Panic_dysf_thought)
	**Somatic symptoms** [6]	“Do you have persistent or recurring pain?” (Somatic_sympt)
	**Hypochondriasis** [1]	“Are you often afraid you might be seriously ill?” (Hypochondriasis)
	**Anorectic symptoms (EATA):** restricted eating [1], omitting meals [2], specific time frames for meals [2], counting calories [1], purging behavior [3], exessive exercise [1], fear of weight gain [1] or loss [1], body weight or shape based self-worth [1]	“Have you set up certain rules, prohibitions, or patterns around eating?” (EATA_restricted) “Do you regularly use laxatives (including natural laxatives)?” (EATA_compens_beh) “Does your weight or body shape determine in a significant way how much you value yourself?” (EATA_body_selfworth)
	**Binge eating and bulimic symptoms (EATB):** loss of control over eating behavior [2], eating notably more than usual [1], intrusive thoughts on eating [1], socially disguised eating behavior [3], eating to cope with negative affect [1], worries around calorie intake and body weight [2]	“Do you often feel like you lose control over what and how much you eat?” (EATB_loss_ctrl) “Have you recently eaten significantly more at times than you wanted to?” (EATB_overeating)
	**Obssessive compulsive disorder (OCD) symptoms** [3]	“Do you experience recurring, irrational, or senseless thoughts that compel you to such actions as repeatedly washing your hands or checking over and over again that the stove is off?” (OCD)
	**Substance use** (drugs [3], alcohol [3])	“Do you use illegal drugs or other medications such as stimulants, sedatives, or prescription-only painkillers without following a doctor’s prescription?” (Subst_abuse_drugs)
	**Dissociation** [6]	“Do you sometimes get the sense you’re seeing others or things as if from very far away or through a fog?” (Dissociation)
	**Psychotic symptoms** [3]	“Do you sometimes hear voices telling you to do things or commenting on what you’re doing?” (Psychotic_sympt)
**Maladaptive traits**	**Negative affectivity (NA):** separation insecurity [3], hostility [1], perseveration [2]	“After a separation or loss, do you tend to feel like you’ve lost your grounding?” (NA_sep_insecur)
	**Detachment (DT):** anhedonia [2], intimacy avoidance [3], restricted affectivity [3], withdrawal [2], suspiciousness [1]	“Do you usually prefer to keep romantic feelings out of your life?” (DT_intim_avoid)
	**Psychoticism (PS):** eccentricity [2], perceptual dysregulation [2], unusual beliefs [2]	“Do you often have thoughts that make sense to you but are strange to other people?” (PS_eccentricity)
	**Disinhibition (DI):** impulsivity [2], irresponsibility [2], norm-violation [1], risk-taking [2], perfectionism [2]	“Do you enjoy new, thrilling things, even if they are against the law?” (DI_normviolation)
	**Antagonism/Dissociality (AN):** callousness[2], deceitfulness [2], entitlement [1], grandiosity [1], manipulativeness [2]	“Do you usually not care if your actions hurt others?” (AN_callousness)
**Personality functioning**	Affect communication [2]	“Do you find it difficult to make others understand you?” (PF_affect_comm)
	Affect differentiation [2]	“Are your feelings often so chaotic you have a hard time describing them?” (PF_affect_diff)
	Affect tolerance [2]	“Are your feelings sometimes so intense that you get scared?” (PF_affect_tolrnce)
	Anticipating behavior of others [1]	“Do you sometimes misjudge how your behavior affects others?” (PF_anticipation)
	Forming relationships [2]	“Do you find it hard to connect with other people?” (PF_forming_relshps)
	Holistic perception of others [2]	“Do you often experience people as either being on the same wavelength as you or absolutely not?” (PF_holistic_percptn)
	Identity [4]	“Do you sometimes feel like a stranger to yourself?” (PF_identity)
	Internal model of relationships [4]	“Is your experience that trusting people too much can bring bad surprises?” (PF_intern_relshp_mod)
	Regulation of self-esteem [3]	“Do you find it difficult to move on quickly after you have been criticized?” (PF_self_est_reg)
	Self reflection [2]	“Do you tend to get confused when you think too much about yourself?” (PF_self_reflection)
	Impulse regulation [3]	“Are you sometimes so full of rage you might lose control?” (PF_impulse_reg)

Domains/disorder categories are in bold, number of items for the respective construct are in brackets.

We averaged all psychopathology scores within participants across 10 months prior to the subsequent analyses. Using multiple averaged assessments yields indicators for stable dispositions and minimizes measurement error [49]. Unidimensionality of all scales with at least two items was ascertained using parallel analysis followed by reliability analyses using McDonald’s ω [50]. Due to the adaptive testing algorithm implemented in the app, the number of available assessments differed substantially per scale. For example, scale hopelessness had on average 13 assessments (range 0 to 61, *SD* = 12.1) whereas others such as symptoms of obsessive compulsive disorder had on average 2.1 (range 0 to 7.3, *SD* = 1.53). Descriptive statistics, hierarchical McDonald’s ω, and number of pairwise complete observations for all 98 psychopathology scales can be found in the Supplemental Material.

#### Assessment of psychosocial impairment

Psychosocial impairment was assessed based on two broad areas (i.e., well-being and basic functioning) initially identified through a joint factor analysis of measures of quality of life, social functioning, and disability [51]. Well-being was captured using items assessing self-acceptance (“Have you been satisfied with yourself lately?”), social relations (“Is the way you’re feeling interfering with how you’re interacting with others?”), and purpose in life (“Are you spending your time on things that are meaningful to you?”). Basic functioning was captured using items assessing mobility (”Is your anxiety or another emotional issue making it difficult for you to leave the house alone or keep appointments?”), self-care (“Are you finding it difficult to maintain your personal hygiene such as taking a shower or brushing your teeth?”), and work/school (“Are you finding it difficult to take care of your responsibilities because of how you feel?”). The average number of available assessments of psychosocial impairment over two years was 78.8 (range 3 to 916, SD = 85.1).

#### Assessment of affective well-being

App users were asked to rate their current mood at each assessment point up to three times daily using a single-item bipolar mood rating scale. Moreover, users were allowed to give mood ratings at any time. The scale consisted of 5 emojis representing different emotions (see Fig. 1). The selected emojis were converted to numeric values ranging from 0 to 4, with 0 indicating the lowest affective well-being at that moment. In a previous study, averaged affective well-being assessments over 2 weeks were a significant indicator of psychopathology [38]. The average number of available momentary affective well-being assessments per user in the study sample over two years was 336.8 (range 20 to 2627, *SD* = 449.0).

### Data analysis

#### Research question 1: integrating personality functioning into HiTOP

To determine the hierarchical structure of psychopathology scales in our data, we applied a bass-ackwards procedure following Forbes et al. [34, 52], using orthogonal equamax rotation. Due to the two-step answer format in the MindDoc app, scales were expected to be zero-inflated (i.e., non-normally distributed), rendering Pearson or Spearman correlations unsuitable for the estimation of the correlation matrix. We therefore applied semi-parametric latent Gaussian copula models [53] for estimation and used the resulting correlation matrix for subsequent analyses with pairwise complete observations. Equamax rotation adjusts for the number of rotated factors, distributing the number of scales with high loadings more evenly between factors than varimax rotation does. This in turn is an important prerequisite for the following bass-ackwards analysis as more general factors are expected to be found in higher levels of the hierarchy. Compared to other rotation methods, equamax is also particularly suitable for reproducing complex data structures [54]. The number of components (n) at the bottom layer of the hierarchy was identified through parallel analysis and Velicer’s minimum average partial (MAP).

#### Research question 2: transdiagnostic variance of personality functioning

To explore which aspects of psychopathology (i.e., scales assessing symptoms, traits, or PF) contribute most to the transdiagnostic variance found in the higher-order components identified in the previous step, we estimated symmetrical bifactor models for every higher-order component. We iteratively specified all higher-order components identified in the previous step as a general (G) factor, and the respective lower-order components loading on this higher-order component as specific (S) factors, using the scales defining these lower-order components as indicators. Parameters and model fit of all bifactor models can be found at https://osf.io/swj3c/. Psychopathology scales with a high G-factor loading and low S-factor loadings (highest in case of scales loading on multiple S-factors) may predominantly capture transdiagnostic variance of the mental health syndromes defining the higher-order component.

#### Research question 3: longitudinal prediction of affective well-being and psychosocial impairment

In case we identified a lower-order component that was both consisting of PF scales (when addressing research question 1) and capturing mainly higher-order or transdiagnostic psychopathology variance (when addressing research question 2), this component would be a candidate for a reference factor in a confirmatory bifactor-(S-1) model [55, 56]. Thus, we aimed to establish a bifactor-(S-1) model with a lower-order component consisting of PF scales as reference factor and all other lower-order components as S-factors, while including longitudinally assessed affective well-being and psychosocial impairment as covariates for all latent factors. We planned to extract the latent covariance matrix of this model and to use it for estimating regression models by means of matrix regression. Using this approach, variance explained (i.e., squared semipartial correlation coefficient, SSPC) in affective well-being and psychosocial impairment by the reference factor (i.e., PF) can be disentangled from unique variance explained by the other factors. To evaluate model fit, we calculated the unbiased SRMR index (SRMRu; [57]) because other common fit indices such as CFI and RMSEA can be biased in scenarios with a large number of variables and a large sample size [58].

## Results

### Hierarchical structure

**Fig. 2:**
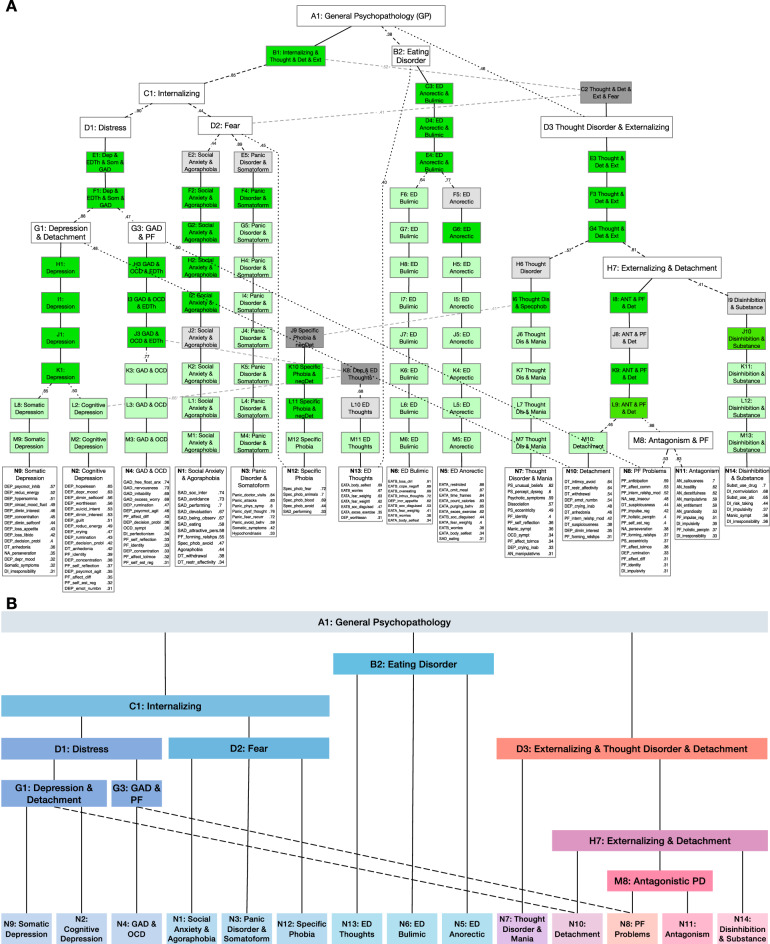
Hierarchical structure of 98 symptoms, traits and PF scales. Complete hierarchical structure of 98 symptoms, traits, and personality functioning with 1–14 components (**A**) and without redundant and artefactual components (**B**) according to Forbes (2023) based on averaged psychopathology data of *N* = 27,173 MindDoc users over 10 months. Note. In **A**, solid lines depict the perpetuation of a component between levels (*r* < 0.9), dashed lines depict emergence of new components (0.3 ≤ |*r* | ≤ 0.9), dotted black lines depict correlations from lower-level to higher-level constructs 0.3 ≤ |*r* | ≤ 0.9 which are not accounted for hierarchically. Redundant components: For constructs that perpetuate to the lowest level of the hierarchy, the version at the bottom of the hierarchy is retained (light green); for constructs that perpetuate from the top or through the middle of the hierarchy, the version of the construct closest to the top is retained (dark green). Components that can be removed due to close-to-redundancy with other components are depicted in light gray. Artefactual constructs that cannot be found in a hierarchical cluster analysis (see Supplementary material) are depicted in dark gray. In **B**, solid lines represent the strongest component correlation for each lower-order component with the higher-order components, and dashed lines are secondary component correlations 0.3 ≤ |*r* | ≤ 0.9.

While Velicer’s minimum average partial (MAP) calculated for 1 to 20 factors reached a minimum with 14 and 15 factors, parallel analysis indicated 14 significant components. Based on these findings, we applied the *ExtendedBassAckwards* function [34], that is, sequential principal component analyses with 1, 2, 3, …, 14 components using equamax rotation combined with hierarchical agglomerative clustering on a latent correlation matrix of 98 psychopathology scales. The resulting hierarchical structure is shown in Fig. 2A, depicting 14 components at the bottom layer with loadings ≥0.31 of 98 scales. Each component was assigned a distinct meaning based on the scales with highest loadings as indicated by the labels used in Fig. 2A. It is important to note that 38 scales exhibited cross-loadings with other lower-order components within one spectrum (e.g., decision problems loading on the two depression subcomponents [N2, N9] and on the generalized anxiety disorder [GAD] subcomponent [N4]). Four PF scales (affect differentiation, affect tolerance, regulation of self esteem, and identity) showed loadings on four different components across spectra.

The final hierarchical structure without redundant and artefactual components is depicted in Fig. 2B. A detailed description of the related bass-ackwards procedure can be found in the supplementary material, reproducible code and all results of the Bass Ackwards procedure can be found at https://osf.io/swj3c/. We found an internalizing higher-order component consisting of fear and distress subcomponents, encompassing cognitive and somatic depression, social anxiety disorder, GAD, agoraphobia, and specific phobia. We also identified an externalizing higher-order component with subcomponents of antagonism, PF, and disinhibition, encompassing antagonistic traits, 10 out of 11 of the PF scales, impulsivity, and substance use problems, as well as a thought disorder lower-level component encompassing schizotypal traits, dissociative, and psychotic symptoms along with OCD and manic symptoms. Somatoform symptoms loaded on both distress and fear subcomponents in the internalizing spectrum. The thought disorder component, along with the externalizing component, formed an externalizing, detachment, and thought disorder superspectrum that loaded on general psychopathology (GP). All three eating disorder (ED)-related lower-order components formed a higher-order ED component that loaded directly on GP. Note that although the lower order PF component primarily loaded on the antagonism subcomponent (M8), it also showed a significant secondary correlation with G3 (GAD & PF). Detachment, while also primarily loading on the externalizing higher-order component, had a significant secondary correlation with G1 (depression).

### Psychopathology scales and components capturing higher-order factor variance

**Fig. 3:**
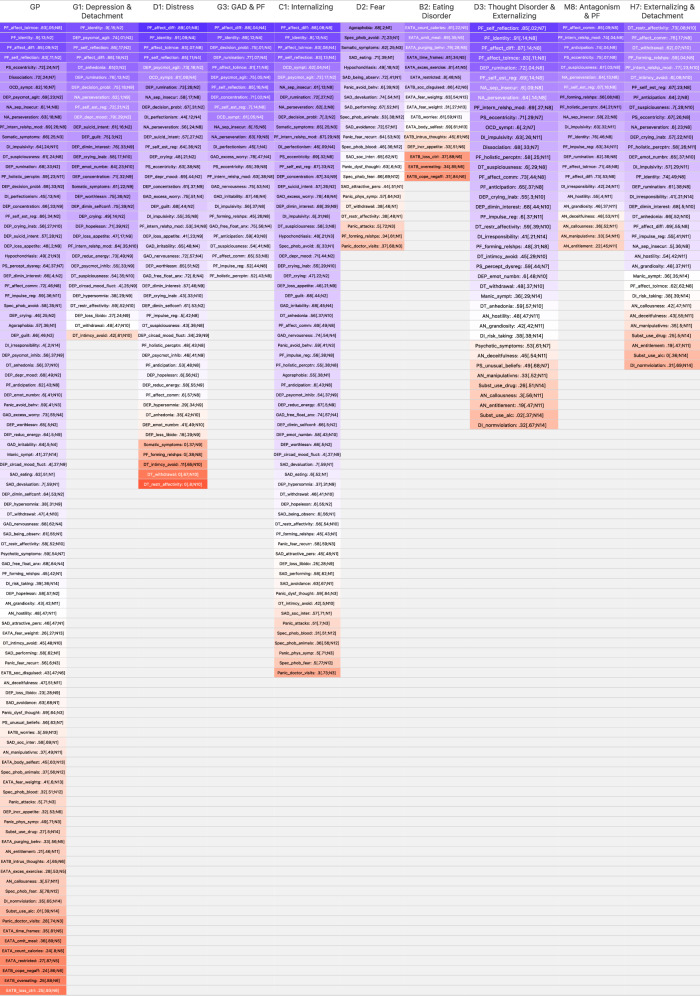
Standardized factor loadings on G and S factors for every higher-order factor from Fig. 2. Standardized loadings on G factor for every scale from separate bifactor models defined for each higher-order factor depicted in the column header and its respective lower-order components as specific factors. Highest loading on specific factors in brackets. Scales in red have higher loadings on specific factors scales in blue have higher loadings on the respective general factor.

All 98 scales were investigated regarding their utility in capturing higher-order factor variance using symmetrical bifactor models for every higher-order component (see https://osf.io/swj3c/ for a reproducible script and model parameters). For instance, the bifactor model for the distress (D1) component included specific factors of somatic depression (N9), cognitive depression (N2), and GAD + OCD (N4), and all indicators of these lower-order components (see Fig. 2) also loaded on the general factor. Figure 3 presents standardized loadings on the G-factor and S-factors (highest loading in case of scales loading on multiple S-factors) of all scales and bifactor models, ordered by the difference between G- and S-loadings. Scales higher on the list (blue color) indicate a stronger association with the common variance of mental health syndromes in the respective higher-order component. Scales in red exhibit higher loadings on specific factors (i.e., lower-order components) than on the general factor (i.e., higher-order component). For most of the higher-order components, including GP, PF scales had the highest loadings on the general factor and lowest loadings on specific factors. Identity problems, affect differentiation, and affect tolerance were most indicative of internalizing disorders (G1, D1, G3, C1). Negative internal relationship models, affect communication, restricted affectivity, and anticipating behavior of others were most indicative of externalizing disorders (H7, M8). Self-reflection, identity, and affect tolerance were most indicative of the thought disorder and externalizing higher-order component (D3). Differential loadings between spectra were found for the detachment facets withdrawal, restricted affectivity, and intimacy avoidance, which were indicators of the general factors for thought disorder and externalizing while showing mainly S-loadings within higher-order internalizing components (G1, D1). Specific phobia and agoraphobia were most indicative of the fear (D2) component, whereas excessive exercising and counting calories were most indicative of the eating disorder (B2) component.

**Fig. 4:**
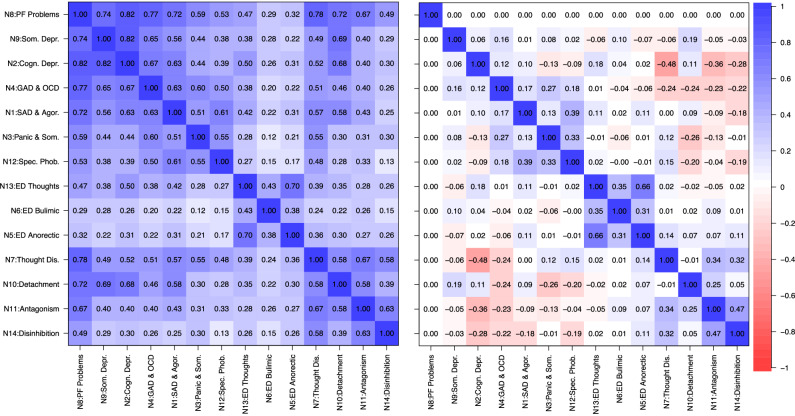
Correlations between lower order components with and without PF variance. Correlations between the 14 lower-order components, modeled in a correlated factors model without cross-loadings of PF scales (left); correlations between residuals of the 13 non-PF lower-order components when variance from PF/N8 component is removed, modeled in a bifactor-(S-1) model (right).

Results from the bifactor models showed that PF scales seem to be pure markers of most of the higher-order components including GP. PF can therefore serve as a reference factor in a bifactor-(S-1) model by partialling out the variance in the other lower-order components that covaries with PF problems [56, 59]. To illustrate this procedure, Fig. 4 depicts latent correlations of a correlated factors model of all lower-order components on the left (without cross-loadings of PF scales), and a bifactor-(S-1) model on the right (with the PF factor N8 as reference, i.e., setting all 13 non-PF factors orthogonal to the PF/N8 factor). The correlated factors correlogram (left panel) shows that the PF/N8 factor is highly correlated with factors from both the internalizing and externalizing spectra (highest average intercorrelation), whereas the correlogram of the bifactor-(S-1) model (right panel) shows that the correlation pattern between all lower-order factors, which is the basis of the higher-order factors found in the previous step, changes substantially if the common variance between the 13 non-PF factors and the PF factor is removed/partialled out. Furthermore, while there are still low to moderately correlated clusters of distress, fear, eating, externalizing, and thought disorder psychopathology, correlations between internalizing and externalizing/thought disorder factors become negative.

### Longitudinal prediction of affective well-being and psychosocial impairment

Table 2 shows a prediction of average psychosocial impairment and affective well-being of users during their first year of app usage including the 10 months of psychopathology assessment (left) and average psychosocial impairment and affective well-being starting 10 months after the first assessment up to two years (right) using regression coefficients and squared semipartial correlations. For the reference factor PF problems (N8), squared semipartial correlations reflect the variance overlap with the criterion while for the remaining specific factors of the bifactor-(S-1) model, they reflect the unique variance explained in the criterion beyond the reference factor and all other specific factors.

**Table 2:** Multiple regression and semipartial correlations based on covariance matrix from bifactor-(S-1) model.

	Prediction 1: Year one				Prediction 2: Year two			
	Psychosocial impairments		Affective well-being		Psychosocial impairments		Affective well-being	
	Std. regr. weights	SSPC	Std. regr. weights	SSPC	Std. regr. weights	SSPC	Std. regr. weights	SSPC
N8:PF Problems	0.77 [0.76, 0.77]	.586	−0.55 [−0.56, −0.55]	.306	0.58 [0.57, 0.58]	.331	−0.48 [−0.49, 0.58]	.232
N9:Som. Depr. (resid.)	0.34 [0.33, 0.34]	.1	−0.12 [−0.13, −0.11]	.013	0.29 [0.28, 0.3]	.074	−0.1 [−0.11, 0.29]	.009
N2:Cogn. Depr. (resid.)	0.34 [0.34, 0.35]	.077	−0.52 [−0.53, −0.51]	.178	0.21 [0.2, 0.22]	.028	−0.36 [−0.38, 0.21]	.086
N4:GAD & OCD (resid.)	−0.02 [−0.02, −0.01]	0	0.01 [0, 0.02]	0	−0.1 [−0.11, −0.09]	.008	0.06 [0.05, −0.1]	.003
N1:SAD & Agor. (resid.)	0.08 [0.08, 0.09]	.005	0.01 [0, 0.02]	0	0.07 [0.06, 0.08]	.004	0.02 [0.01, 0.07]	0
N3:Panic & Som. (resid.)	0.01 [0.01, 0.02]	0	.[−0.01, 0.01]	0	−0.02 [−0.03, −0.01]	0	0.05 [0.04, −0.02]	.002
N12:Spec. Phob. (resid.)	0.[−0.01, 0]	0	−0.02 [−0.03, −0.01]	0	0.03 [0.02, 0.04]	0	−0.04 [−0.05, 0.03]	.001
N13:ED Thoughts (resid.)	0.[0, 0.01]	0	0.08 [0.07, 0.09]	.003	0.02 [0, 0.03]	0	0.06 [0.04, 0.02]	.002
N6:ED Bulimic (resid.)	0.01 [0.01, 0.02]	0	.[−0.01, 0.01]	0	0.03 [0.02, 0.04]	.001	−0.01 [−0.02, 0.03]	0
N5:ED Anorectic (resid.)	−0.01 [−0.02, 0]	0	−0.07 [−0.08, −0.06]	.002	−0.02 [−0.03, 0]	0	−0.07 [−0.08, −0.02]	.003
N7:Thought Dis. (resid.)	−0.01 [−0.02, 0]	0	−0.1 [−0.11, −0.1]	.007	−0.03 [−0.04, −0.02]	0	−0.06 [−0.07, −0.03]	.002
N10:Detachment (resid.)	0.03 [0.02, 0.03]	0	−0.14 [−0.15, −0.14]	.015	0.01 [0, 0.02]	0	−0.12 [−0.13, 0.01]	.011
N11:Antagonism (resid.)	0.02 [0.02, 0.03]	0	0.02 [0.01, 0.03]	0	0.02 [0.01, 0.03]	0	0.03 [0.02, 0.02]	.001
N14:Disinhibition (resid.)	0.01 [0.01, 0.02]	0	0.03 [0.03, 0.04]	.001	−0.05 [−0.06, −0.04]	.001	0.08 [0.07, −0.05]	.004

N (affective well-being prediction 1) = 25844. N (affective well-being prediction 2) = 10636. N (psychosocial functioning prediction 1) = 27173. N (psychosocial functioning prediction 2) = 5342. Total R2 affective well-being (prediction 1) = 0.614. Total R2 psychosocial functioning (prediction 1) = 0.847. Total R2 affective well-being (prediction 2) = 0.424. Total R2 psychosocial functioning (prediction 2) = 0.487. SSPC = squared semipartial correlation, i.e. the unique contribution of a specific predictor variable in a multiple regression analysis, while controlling for other predictors in the model.

Regarding the prediction of averaged impairment in psychosocial functioning in the first year, PF accounted for 58.6% of 84.7% total variance explained, with variance specific to somatic (10.0%) and cognitive depression (7.7%), as well as social anxiety (0.5%) also contributing significantly in the multiple regression models. Concerning averaged affective well-being in the first year, PF accounted for 30.6% of 61.4% total variance explained with variance specific to cognitive depression symptoms (17.8%), somatic depression symptoms (1.3%), thought disorder (0.7%), and detachment (1.5%) significantly contributing as well. Predicting averaged impairment in psychosocial functioning from 10 months up to two years after the first assessment, PF accounted for 33.1% of 48.7% total variance explained with cognitive (2.8%) and somatic depression (7.4%) symptoms and GAD (0.8%) contributing significantly. Concerning averaged affective well-being between 10 and 24 months after the first assessment, PF accounted for 23.2% of 42.4% total variance explained with variance specific to cognitive (8.6%) and somatic (0.9%) depression symptoms and detachment (1.1%) contributing significantly. Reproducible code for bifactor-(S-1) modeling and prediction can be found at https://osf.io/swj3c/.

## Discussion

The aim of this study was to investigate the role of PF within the HiTOP framework of psychopathology using ambulatory assessed longitudinal data over two years in a sample of *N* = 27,173 mental health app users. We conducted a bass-ackwards analysis that yielded a hierarchical taxonomy of psychopathological symptoms, traits, and PF (research question 1), which we subsequently used for latent modeling of general and specific component variance (research question 2) and longitudinal prediction (research question 3). Using a very large sample with repeated measurements, this study achieves an unprecedented level of measurement and estimation accuracy [49] with respect to answering the present research questions.

### Locating personality functioning in a hierarchical dimensional structure of psychopathology

In our sample, we replicated a hierarchical dimensional structure that largely aligns with the HiTOP model [3]. However, we identified a distinct PF component that was indicative of internalizing, externalizing, and general psychopathology. Our findings also support previous evidence on higher-order constructs, including an internalizing spectrum with subfactors of distress and fear, a thought disorder component with psychotic symptoms, manic symptoms and schizotypal traits, and an externalizing spectrum with antagonistic and disinhibited components. OCD symptoms showed comparable loadings both on the thought disorder and generalized anxiety components corroborating previous evidence on the association of OCD with both fear and thought disorder related aspects [60]. Further, we found depression to split into a somatic and a cognitive component on the lower level. This finding aligns with several studies [61–63] detecting the distinction between cognitive and somatic depression symptoms to be helpful in the prediction of inflammation, coronary syndrome and HPA-axis hyperactivity. Divergences were found with respect to the eating disorder higher-order component, which only had low to moderate correlation with the Internalizing higher-order component. Nevertheless, its subfactor structure encompassing restrictive, bulimic and well-being-related eating disorder components was already found previously [64]. Moreover, the detachment component exhibited stronger correlations with the externalizing spectrum, especially with antagonism, as well as with (somatic) depression symptoms than with the thought disorder spectrum. However, several other studies also found a detachment component emerging from a higher-order externalizing factor [65, 66].

Most notably, 10 of the 11 PF scales formed a distinct PF component (N8) with the PF facets *anticipating behavior of others*, *affect communication* and *internal model of relationships* showing the highest loadings. These scales were highly indicative of the *p*-factor in Wendt et al. (2023), and all PF scales loading on the PF component were highly indicative of a general factor of personality functioning in another study [40]. In addition, we found small to moderate loadings of *separation insecurity*, *suspiciousness*, *perseveration*, *eccentricity*, and *impulsivity* on this component. Most of these trait scales have moderate to large correlations with the total score or subdomains of DSM-5 PF [31]. These previous findings indicate that the N8 component found in our study mainly captures variance that is due to PF.

Taken together, while our findings corroborate previous evidence on empirical covariation of psychopathological syndromes, i.e. the comorbidity problem, which led to the development of HiTOP in the first place, they also point towards PF as a construct that is identifiable as a distinct component which shows moderate to high correlations across spectra and hierarchical levels.

### Personality functioning as a transdiagnostic construct capturing higher-order component variance and predicting future outcomes

The use of symmetrical bifactor models to identify central indicators for higher-order factors in combination with a bifactor-(S-1) model for assessing the predictive validity of PF compared to other residualized lower-order components were inspired by suggestions on “riskier tests” and “bringing theory to the fore” concerning research on higher-order factors of psychopathology [67, 68]. Using this exploratory approach, several PF indicators such as *identity*, *affect differentiation*, *self reflection*, *affect tolerance*, *internal relationship models*, and *affect communication* were identified to be pure markers of the internalizing, thought disorder, and externalizing spectra. Whereas for the externalizing spectrum, more interpersonal aspects of PF (*affect communication, anticipation, internal model of relationships*) defined the higher-order factor, for the internalizing spectrum, more self-related aspects of PF (*identity, affect differentiation, self reflection, affect tolerance*) defined the higher-order factor. Most of these scales that capture what could also be called “mentalizing impairments regarding one’s own mental states” were previously described as “pure markers of p” [33]. Our findings therefore suggest that aspects of PF play a significant role in a broad range of mental disorders. This is in line with the underpinnings of PF according to psychodynamic etiological theory as summarized by Bender and colleagues [14]: “Biological and environmental problems and their interactions can lead to maladaptive mental models of self and others, and to maladaptive patterns of emotional experience and expression, cognition, and behavior. These, in turn, may lead to the development of psychopathology in general and personality pathology in particular” (p. 344).

The basis for the higher-order structure, that is, the high correlation between the 14 factors on the bottom layer, seems to change significantly if variance that is attributable to PF is partialed out using a bifactor-(S-1) approach. While small to moderately correlated clusters of eating, fear, distress, externalizing, and thought disorder still remain, correlations between residualized components of internalizing and externalizing spectra are negative after partialling out PF. This indicates that PF may explain substantial parts of the variance of higher-order constructs in HiTOP. It could also indicate why patients with different disorders share common etiological pathways and respond to the same treatments. While this hypothesis cannot be corroborated using models of covariation, it is tentatively supported by the longitudinal prediction of affective well-being and psychosocial impairment using PF and residualized lower-order components in our sample. Specifically, in the longer run (up to two years), PF accounted for more than two-thirds of total variance explained in psychosocial impairment and more than half of the total variance explained in affective well-being in multiple regression models including 13 additional predictors based on 87 psychopathology scales.

### Clinical implications

Following our findings of the importance of PF in a hierarchical taxonomy of psychopathology, clinicians may assess *identity problems*, *affect differentiation*, and *communication*, along with *internal relationship models*, as etiologically informed and prognostically relevant indicators for a broad range of mental disorders. A number of well-validated PF measures may thus provide both parsimonious and reliable utility for assessment and treatment planning. This is in line with recent practical recommendations [69–71] which emphasize that adaptations of treatment indication, modality, and intensity may be based on individual PF assessments. Drawing on findings of PF impairments and their clinical relevance (e.g., higher drop-out rates, less therapy compliance, more risk for ruptures in the therapeutic relationship, and generally higher rates of comorbidity and chronicity), patients with mild impairments in PF may need comparably less intense or structured treatments, whereas patients with moderate and high PF impairments may need highly structured settings and more intense or process-oriented treatments, with a particular emphasis on reducing destructive tendencies towards the self and others [69]. Our findings also tentatively suggest that treatment of internalizing disorders may focus on self aspects of PF, while treatment of externalizing disorders may focus predominantly on interpersonal aspects of PF. We therefore suggest the inclusion of short measures for PF, such as LPFS-BF 2.0 [72] or OPD-SQS [43], and maladaptive traits, such as the PID5BF + M [73], in routine outcome monitoring. For time-limited settings, a recent study suggests focusing on specific subdomains of intimacy and identity to clinically approximate PF [74].

### Directions for future research

First, research should investigate PF as a potential treatment indicator and target change in transdiagnostic PF features as outcome. While long-term interventions such as psychotherapy seem to be effective for changing PF [75] and traits [22, 76], future research is needed to differentiate the impact on different PF facets. In addition, the development of scalable (digitally aided) interventions that help to support change in PF may be relevant for general healthcare.

Secondly, research on etiological processes should investigate links to transdiagnostic PF features [77]. Disentangling transdiagnostic from specific variance using psychometrically sound latent constructs could also advance studies on genetic mechanisms in psychopathology [8, 78]. Furthermore, longitudinal research that explores the development of PF from early childhood to young adulthood up to adult age with concurrent and HiTOP-conform assessment of psychopathology and allostatic load [79] over a long period of time is needed to investigate etiological questions of causality. A useful statistical approach to disentangle “surface characteristics” from “core processes” [80] could be longitudinal bifactor-(S-1) models. This approach could also be applied to repetitive assessments of psychopathology and PF within an EMA framework. Future developments in mental health tracking apps should implement strategies to provide low time gaps between assessments to enable within-person dynamic aspects of HiTOP using dynamic structural equation modeling [81].

Finally, in addition to data-driven approaches such as HiTOP, it is also important to continue developmentally informed, theory-based, and theory-oriented research on psychopathology [24, 82–84]. Integrating personality functioning into these models can help to broaden existing approaches, with its descriptive approach on capacities helping to balance or integrate more specific conceptualizations. In the long term, it may also shed light on important empirical and conceptual questions regarding the *p*-factor [68]. Although progress has been made in differentiating symptoms and traits in the HiTOP model [11], further differentiation with regard to personality functioning is needed. The results of the current study supports other studies that argue that personality functioning may indeed not just index different ways of expressing maladaptive traits [31]. At the same time, the general HiTOP approach has the potential of including these different perspectives into one empirically based model.

### Limitations

A number of limitations should be taken into account when interpreting the results of the current study. Approximations on sample characteristics can only be obtained from a separate assessment of a subsample of MindDoc users (see https://osf.io/swj3c/) and a previous clinical trial [36]. The scales or components of psychopathology that were used for the bass-ackwards-procedure differed with respect to their level of abstraction. For example, the schizophrenia spectrum was assessed with one scale whereas eating pathology was assessed with a total of 14 scales and other areas of interest such as PTSD were missing completely due to the given conceptualization of the mental health app. Further, the selection of items and the labeling of constructs used in this study may be subject to “jingle-jangle-fallacies” [85]. While both the MindDoc team and authors of this study examined redundancy in item content concerning PF, traits and symptoms, it may still be objected that PF and one of the outcomes, psychosocial impairment, cannot be separated conceptually, i.e. different labels for the same phenomenon. However, items used in this study to assess psychosocial impairments were aligned with constructs previously shown to be separable of PF in terms of exploratory factor analysis [51]. Furthermore, the two-step answer format leads to high skewness. Although we were able to address this with using latent correlations, and the extracted hierarchical structure was similar to other studies using different methods, we cannot rule out consequences regarding our results. In addition, some artefactual components that were removed in the bass-ackwards procedure represent clinically valid phenomena. For example, a patient with predominant anorexic or bulimic features could primarily seek help because of depressive symptoms (K8).

Methodological issues may concern the assessments based on self-reports and the accuracy of stepwise estimation using correlation matrices. Furthermore, causal etiological conclusions cannot be drawn unambiguously from our data as the bass-ackwards analysis was based on longitudinally averaged indicators and covariation does not automatically imply a common cause for disorders [86]. However, using repetitive longitudinally averaged assessments in a very large sample both removes confounding of within- and between-person variance and reduces random measurement error, both of which represent key shortcomings of cross-sectional self-report data [49]. We could also demonstrate substantial effects of PF on outcomes in longitudinal prediction. Furthermore, the two main outcomes on prediction differed in assessment methodology as affective well-being was assessed through ambulatory assessment (three times per day) and psychosocial impairment included reverse coded items.

## Conclusion

Our findings can be interpreted as an empirical confirmation of the assumption that PF, including problems of identity, internal models of relationships, self-reflection, emotion awareness, and regulation, lies at the core of psychopathology. The extent to which these psychological capacities are a result of early childhood gene-environment interactions, as initially predicted by psychodynamic and interpersonal theories, and whether they may serve as a fruitful target of transdiagnostic mental health interventions is subject to future studies. However, disentangling transdiagnostic and specific variance in behavioral assessments of psychopathology may be crucial for advancements in all areas of psychiatry.

### Supplementary information

Supplementary Material 1

Supplementary Material 2

Supplementary Material 3

## Data Availability

The latent correlation matrix including R markdown code generating all tables and figures of this paper are available as open data and code via the Open Science Framework repository: https://osf.io/swj3c/.
