# Supplementary material for: Examining the role of personality functioning in a hierarchical taxonomy of psychopathology using two years of ambulatory assessed data
Source: Transl Psychiatry. 2024 Aug 24;14:340. doi: 10.1038/s41398-024-03046-z (PMC11344763; doi:10.1038/s41398-024-03046-z)
Supplement: Supplementary file 1 — Supplementary Material 1 [file 41398_2024_3046_MOESM1_ESM.docx]

## Supplemental material 1: Assessment frequency, scale descriptives, reliability

### Figure. Retention of *N* = 27 173 users within the two-year period of data assessment underlying this study.


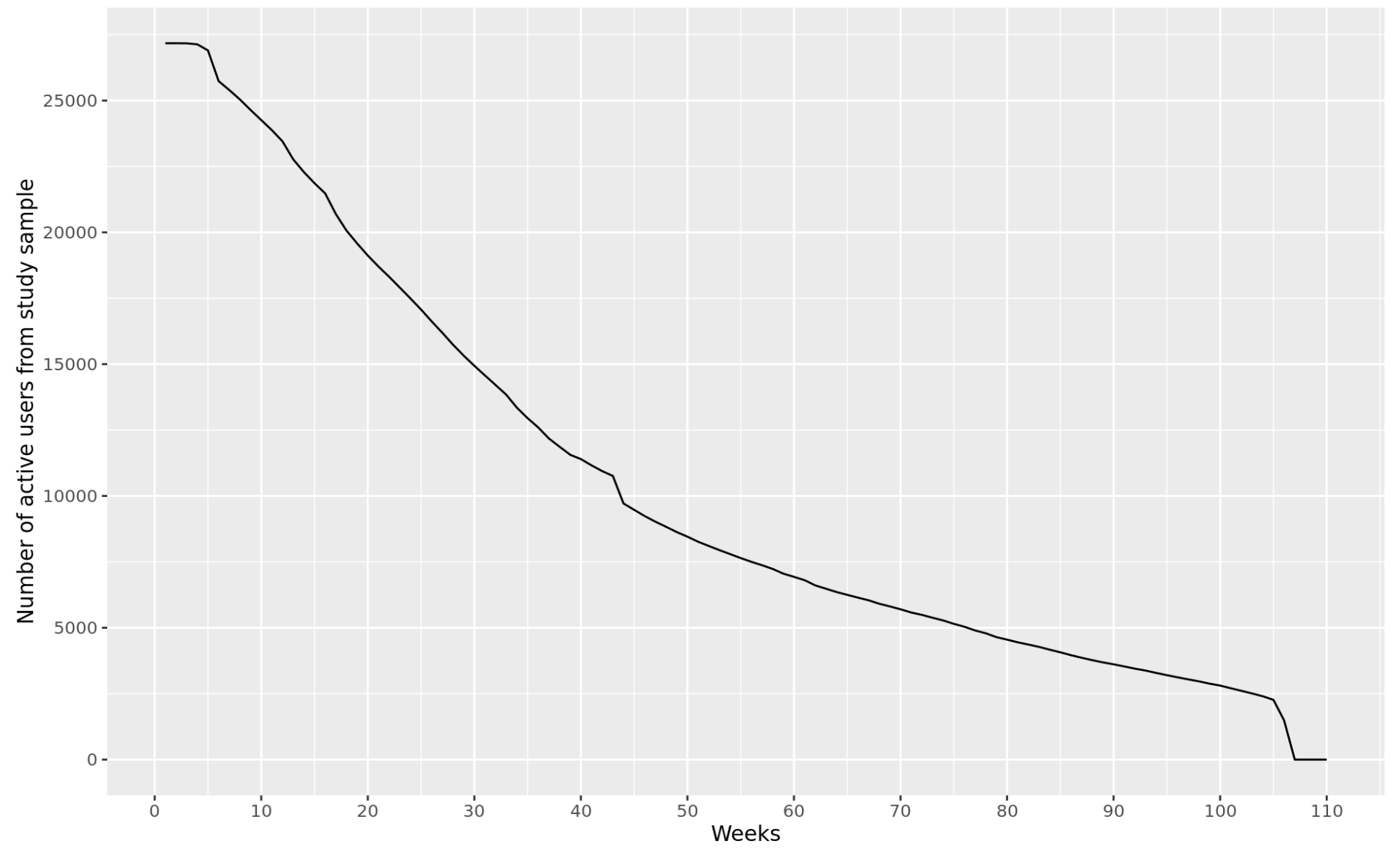


###

### Table. Average frequency of assessment per scale

| scale | min | max | sd | mean |
| --- | --- | --- | --- | --- |
| Agoraphobia (Agoraphobia) | 0.00 | 7.29 | 0.77 | 0.75 |
| AN_callousness (Antagonism: Callousness) | 0.00 | 11.00 | 2.73 | 3.23 |
| AN_deceitfulness (Antagonism: Deceitfulness) | 0.00 | 11.50 | 2.72 | 3.23 |
| AN_entitlement (Antagonism: Entitlement) | 0.00 | 12.00 | 2.74 | 3.24 |
| AN_grandiosity (Antagonism: Grandiosity) | 0.00 | 12.00 | 2.74 | 3.23 |
| AN_hostility (Antagonism: Hostility) | 0.00 | 12.00 | 2.74 | 3.24 |
| AN_manipulativns (Antagonism: Manipulativeness) | 0.00 | 11.00 | 2.72 | 3.23 |
| DEP_circad_mood_fluct (Depression: Circadian mood fluctuations) | 0.00 | 55.00 | 10.90 | 10.04 |
| DEP_concentration (Depression: Concentration difficulties) | 0.00 | 42.00 | 8.28 | 9.01 |
| DEP_crying (Depression: Crying) | 0.00 | 56.00 | 10.93 | 10.04 |
| DEP_crying_inab (Depression: Crying inability) | 0.00 | 60.00 | 10.55 | 9.36 |
| DEP_decision_probl (Depression: Decision problems) | 0.00 | 65.00 | 13.87 | 15.80 |
| DEP_depr_mood (Depression: Depressed mood) | 0.50 | 52.50 | 9.83 | 10.46 |
| DEP_dimin_interest (Depression: Diminished interest in activities) | 0.33 | 49.33 | 9.97 | 10.19 |
| DEP_dimin_selfconf (Depression: Diminished self confidence) | 0.00 | 55.50 | 11.64 | 12.40 |
| DEP_emot_numbn (Depression: Feeling of emotional numbness) | 0.00 | 58.00 | 11.64 | 11.72 |
| DEP_guilt (Depression: Feelings of guilt) | 0.00 | 56.00 | 11.66 | 11.74 |
| DEP_hopelessn (Depression: Hopelessness) | 0.00 | 55.33 | 11.97 | 13.09 |
| DEP_hypersomnia (Depression: Hypersomnia) | 0.00 | 57.00 | 11.09 | 10.22 |
| DEP_incr_appetite (Depression: Increased appetite) | 0.00 | 56.00 | 11.07 | 10.20 |
| DEP_loss_appetite (Depression: Loss of appetite) | 0.00 | 56.00 | 11.10 | 10.26 |
| DEP_loss_libido (Depression: Loss of sexual libido) | 0.00 | 58.00 | 11.60 | 11.80 |
| DEP_psycmot_agit (Depression: Psychomotoric agitation) | 0.00 | 61.00 | 12.11 | 13.11 |
| DEP_psycmot_inhib (Depression: Psychomotric inhibition) | 0.00 | 58.00 | 12.56 | 13.32 |
| DEP_reduc_energy (Depression: Reduced energy) | 0.33 | 54.00 | 11.98 | 12.95 |
| DEP_rumination (Depression: Rumination) | 0.00 | 56.00 | 11.34 | 11.37 |
| DEP_suicid_intent (Depression: Suicidal intentions) | 0.00 | 54.00 | 11.15 | 11.96 |
| DEP_worthlessn (Depression: Feelings of worthlessness) | 0.00 | 59.00 | 12.11 | 13.08 |
| DI_impulsivity (Disinhibition: Impulsivity) | 0.00 | 11.00 | 2.73 | 3.23 |
| DI_irresponsibility (Disinhibition: Irresponsibility) | 0.00 | 11.50 | 2.72 | 3.23 |
| DI_normviolation (Disinhibition: Norm-violation) | 0.00 | 11.00 | 2.74 | 3.24 |
| DI_perfectionism (Disinhibition: Perfectionism) | 0.00 | 14.00 | 3.70 | 4.32 |
| DI_risk_taking (Disinhibition: Risk taking) | 0.00 | 11.00 | 2.72 | 3.23 |
| Dissociation (Dissociation) | 0.00 | 15.00 | 1.47 | 1.11 |
| DT_anhedonia (Detachment: Anhedonia) | 0.00 | 17.00 | 3.38 | 3.84 |
| DT_intimcy_avoid (Detachment: Intimacy avoidance) | 0.00 | 15.00 | 3.31 | 3.85 |
| DT_restr_affectivity (Detachment: Restricted affectivity) | 0.00 | 17.00 | 3.57 | 4.07 |
| DT_suspiciousness (Detachment: Suspiciousness) | 0.00 | 12.00 | 2.74 | 3.24 |
| DT_withdrawal (Detachment: Withdrawal) | 0.00 | 17.50 | 3.64 | 4.08 |
| EATA_body_selfest (Anorectic eating disorder: Body weight or shape based self-worth) | 0.00 | 36.00 | 7.31 | 6.43 |
| EATA_count_calories (Anorectic eating disorder: Counting calories) | 0.00 | 22.00 | 1.13 | 0.29 |
| EATA_exces_exercise (Anorectic eating disorder: Exessive exercise) | 0.00 | 32.00 | 2.60 | 0.86 |
| EATA_fear_weightg (Anorectic eating disorder: Fear of weight gain) | 0.00 | 37.00 | 7.59 | 6.60 |
| EATA_fear_weightl (Anorectic eating disorder: Fear of weight loss) | 0.00 | 36.00 | 7.40 | 6.50 |
| EATA_omit_meal (Anorectic eating disorder: Omitting meals) | 0.00 | 22.00 | 1.06 | 0.28 |
| EATA_purging_behv (Anorectic eating disorder: Purging behavior) | 0.00 | 21.33 | 1.68 | 0.97 |
| EATA_restricted (Anorectic eating disorder: Restricted eating) | 0.00 | 14.00 | 2.32 | 1.24 |
| EATA_time_frames (Anorectic eating disorder: Specific time frames for meals) | 0.00 | 32.50 | 2.09 | 0.96 |
| EATB_cope_negaff (Bulimic eating disorder: Eating to cope with negative affect) | 0.00 | 32.00 | 2.67 | 1.10 |
| EATB_intrus_thoughts (Bulimic eating disorder: Intrusive thoughts on eating) | 0.00 | 33.00 | 2.74 | 1.13 |
| EATB_loss_ctrl (Bulimic eating disorder: Loss of control over eating behavior) | 0.00 | 31.00 | 2.20 | 1.11 |
| EATB_overeating (Bulimic eating disorder: Eating notably more than usual) | 0.00 | 14.00 | 2.39 | 1.26 |
| EATB_soc_disguised (Bulimic eating disorder: Socially disguised eating behavior) | 0.00 | 24.33 | 3.94 | 3.65 |
| EATB_worries (Bulimic eating disorder: Worries around calorie intake and body weight) | 0.00 | 33.50 | 6.73 | 6.28 |
| GAD_excess_worry (Generalized anxiety disorder: Excessive worrying) | 0.00 | 14.20 | 2.20 | 2.29 |
| GAD_free_float_anx (Generalized anxiety disorder: Free floating anxiety) | 0.00 | 9.00 | 0.50 | 0.48 |
| GAD_irritability (Generalized anxiety disorder: Irritability) | 0.00 | 9.00 | 0.51 | 0.48 |
| GAD_nervousness (Generalized anxiety disorder: Subjective experience of nervousness) | 0.00 | 9.50 | 0.50 | 0.48 |
| Hypochondriasis (Hypochondriasis) | 0.00 | 25.00 | 3.78 | 4.47 |
| Manic_sympt (Manic symptoms) | 0.00 | 6.67 | 1.47 | 2.02 |
| NA_perseveration (Negative Affectivity: Perseveration) | 0.00 | 15.00 | 3.60 | 4.14 |
| NA_sep_insecur (Negative Affectivity: Separation insecurity) | 0.00 | 17.33 | 4.72 | 5.20 |
| OCD_sympt (Obsessive compulsive disorder symptoms) | 0.00 | 7.33 | 1.53 | 2.09 |
| Panic_attacks (Panic attacks) | 0.00 | 23.50 | 5.78 | 4.34 |
| Panic_avoid_behv (Avoidance behavior due to previous panic attacks) | 0.00 | 23.00 | 2.50 | 1.72 |
| Panic_doctor_visits (Doctor visits due to previous panic attacks) | 0.00 | 6.00 | 0.37 | 0.15 |
| Panic_dysf_thought (Dysfunctional thoughts during panic attacks) | 0.00 | 2.33 | 0.48 | 0.59 |
| Panic_fear_recurr (Fear of recurring panic attacks) | 0.00 | 3.00 | 0.50 | 0.59 |
| Panic_phys_symp (Physical symptoms during panic attacks) | 0.00 | 3.00 | 0.50 | 0.59 |
| PF_affect_comm (Personality functioning: Affect communication) | 0.00 | 20.50 | 4.86 | 5.40 |
| PF_affect_diff (Personality functioning: Affect differentiation) | 0.00 | 21.00 | 5.23 | 5.85 |
| PF_affect_tolrnce (Personality functioning: Affect tolerance) | 0.00 | 20.50 | 5.23 | 5.82 |
| PF_anticipation (Personality functioning: Anticipating behavior of others) | 0.00 | 25.00 | 6.35 | 6.74 |
| PF_forming_relshps (Personality functioning: Forming relationships) | 0.00 | 40.00 | 8.68 | 9.16 |
| PF_holistic_percptn (Personality functioning: Holistic perception of others) | 0.00 | 12.00 | 2.73 | 3.23 |
| PF_identity (Personality functioning: Identity problems) | 0.00 | 19.00 | 4.75 | 5.34 |
| PF_impulse_reg (Personality functioning: Impulse regulation) | 0.00 | 11.00 | 2.72 | 3.23 |
| PF_intern_relshp_mod (Personality functioning: Internal model of relationships) | 0.00 | 23.25 | 6.10 | 6.73 |
| PF_self_est_reg (Personality functioning: Regulation of self-esteem) | 0.00 | 17.00 | 4.38 | 5.01 |
| PF_self_reflection (Personality functioning: Self reflection) | 0.00 | 20.50 | 5.23 | 5.82 |
| PS_eccentricity (Psychoticism: Eccentricity) | 0.00 | 11.50 | 2.72 | 3.23 |
| PS_percept_dysreg (Psychoticism: Perceptual dysregulation) | 0.00 | 11.00 | 2.72 | 3.23 |
| PS_unusual_beliefs (Psychoticism: Unusual beliefs) | 0.00 | 11.00 | 2.72 | 3.24 |
| Psychotic_symptoms (Psychotic symptoms) | 0.00 | 11.00 | 2.72 | 3.23 |
| SAD_attractive_pers (Social anxiety when interacting with attractive persons) | 0.00 | 4.00 | 0.50 | 0.59 |
| SAD_avoidance (Social anxiety: avoidance of interactions with strangers) | 0.00 | 16.00 | 0.49 | 0.34 |
| SAD_being_observ (Social anxiety with doing tasks while being observed) | 0.00 | 3.00 | 0.50 | 0.59 |
| SAD_devaluation (Social anxiety: Fear of devaluation) | 0.00 | 16.00 | 0.48 | 0.34 |
| SAD_eating (Social anxiety when eating in front of others) | 0.00 | 16.00 | 0.49 | 0.34 |
| SAD_performing (Social anxiety when performing in front of others) | 0.00 | 7.50 | 0.46 | 0.47 |
| SAD_soc_inter (Social anxiety in social interactions) | 0.00 | 19.50 | 4.23 | 3.75 |
| Somatic_symptoms (Somatic symptoms) | 0.17 | 17.83 | 3.60 | 4.28 |
| Spec_phob_animals (Specific phobia: Animals) | 0.00 | 3.00 | 0.50 | 0.59 |
| Spec_phob_avoid (Specific phobia: Avoidance of feared objects) | 0.00 | 3.00 | 0.50 | 0.59 |
| Spec_phob_blood (Specific phobia: Blood-injury phobia) | 0.00 | 3.00 | 0.50 | 0.59 |
| Spec_phob_fear (Specific phobia: Excessive fear of certain objects) | 0.00 | 13.00 | 2.64 | 2.58 |
| Subst_use_alc (Substance use: Regular use of alcohol) | 0.00 | 34.67 | 7.21 | 7.41 |
| Subst_use_drug (Substance use: Regular use of drugs) | 0.00 | 20.67 | 5.52 | 5.25 |

Table: Scale descriptives and reliabilities

| scale | n | mean | sd | skew | kurtosis | se | omega |
| --- | --- | --- | --- | --- | --- | --- | --- |
| Agoraphobia (Agoraphobia) | 27033 | 0.79 | 1.15 | 1.26 | 0.35 | 0.01 | 0.82 |
| AN_callousness (Antagonism: Callousness) | 27044 | 0.28 | 0.63 | 2.87 | 8.98 | 0.00 | 0.90 |
| AN_deceitfulness (Antagonism: Deceitfulness) | 27084 | 0.66 | 0.91 | 1.50 | 1.62 | 0.01 | 0.87 |
| AN_entitlement (Antagonism: Entitlement) | 26659 | 0.29 | 0.75 | 2.90 | 8.23 | 0.00 | NA |
| AN_grandiosity (Antagonism: Grandiosity) | 26647 | 0.67 | 1.08 | 1.55 | 1.29 | 0.01 | NA |
| AN_hostility (Antagonism: Hostility) | 26677 | 0.52 | 0.96 | 1.87 | 2.59 | 0.01 | NA |
| AN_manipulativns (Antagonism: Manipulativeness) | 27066 | 0.48 | 0.85 | 2.00 | 3.46 | 0.01 | 0.88 |
| DEP_circad_mood_fluct (Depression: Circadian mood fluctuations) | 25946 | 0.92 | 1.17 | 1.14 | 0.13 | 0.01 | NA |
| DEP_concentration (Depression: Concentration difficulties) | 27164 | 2.04 | 1.25 | -0.17 | -1.12 | 0.01 | 0.82 |
| DEP_crying (Depression: Crying) | 25925 | 0.96 | 1.18 | 1.15 | 0.16 | 0.01 | NA |
| DEP_crying_inab (Depression: Crying inability) | 25099 | 1.06 | 1.26 | 0.95 | -0.41 | 0.01 | NA |
| DEP_decision_probl (Depression: Decision problems) | 27168 | 1.61 | 1.32 | 0.29 | -1.28 | 0.01 | NA |
| DEP_depr_mood (Depression: Depressed mood) | 27173 | 1.85 | 1.10 | 0.15 | -0.94 | 0.01 | 0.84 |
| DEP_dimin_interest (Depression: Diminished interest in activities) | 27173 | 1.53 | 1.21 | 0.41 | -1.06 | 0.01 | 0.93 |
| DEP_dimin_selfconf (Depression: Diminished self confidence) | 27167 | 1.55 | 1.18 | 0.40 | -1.00 | 0.01 | 0.75 |
| DEP_emot_numbn (Depression: Feeling of emotional numbness) | 26766 | 0.98 | 1.24 | 1.06 | -0.21 | 0.01 | NA |
| DEP_guilt (Depression: Feelings of guilt) | 26749 | 1.59 | 1.34 | 0.32 | -1.26 | 0.01 | NA |
| DEP_hopelessn (Depression: Hopelessness) | 27172 | 1.24 | 1.25 | 0.77 | -0.73 | 0.01 | 0.93 |
| DEP_hypersomnia (Depression: Hypersomnia) | 25926 | 0.78 | 1.01 | 1.43 | 1.22 | 0.01 | NA |
| DEP_incr_appetite (Depression: Increased appetite) | 25977 | 0.65 | 1.05 | 1.77 | 2.21 | 0.01 | NA |
| DEP_loss_appetite (Depression: Loss of appetite) | 25971 | 0.55 | 0.85 | 1.96 | 3.54 | 0.01 | NA |
| DEP_loss_libido (Depression: Loss of sexual libido) | 27037 | 0.91 | 1.16 | 1.23 | 0.39 | 0.01 | NA |
| DEP_psycmot_agit (Depression: Psychomotoric agitation) | 27115 | 1.73 | 1.27 | 0.17 | -1.23 | 0.01 | NA |
| DEP_psycmot_inhib (Depression: Psychomotric inhibition) | 27090 | 0.64 | 0.98 | 1.71 | 2.09 | 0.01 | NA |
| DEP_reduc_energy (Depression: Reduced energy) | 27173 | 1.84 | 1.06 | 0.20 | -0.98 | 0.01 | 0.89 |
| DEP_rumination (Depression: Rumination) | 27002 | 1.94 | 1.34 | -0.03 | -1.33 | 0.01 | NA |
| DEP_suicid_intent (Depression: Suicidal intentions) | 26796 | 0.88 | 1.15 | 1.27 | 0.47 | 0.01 | NA |
| DEP_worthlessn (Depression: Feelings of worthlessness) | 27107 | 1.26 | 1.36 | 0.71 | -0.95 | 0.01 | NA |
| DI_impulsivity (Disinhibition: Impulsivity) | 27070 | 1.01 | 1.12 | 0.93 | -0.23 | 0.01 | 0.80 |
| DI_irresponsibility (Disinhibition: Irresponsibility) | 27073 | 0.45 | 0.76 | 1.97 | 3.74 | 0.00 | 0.80 |
| DI_normviolation (Disinhibition: Norm-violation) | 26651 | 0.56 | 1.00 | 1.78 | 2.19 | 0.01 | NA |
| DI_perfectionism (Disinhibition: Perfectionism) | 27146 | 1.01 | 1.16 | 0.94 | -0.29 | 0.01 | 0.83 |
| DI_risk_taking (Disinhibition: Risk taking) | 27071 | 0.51 | 0.82 | 1.95 | 3.62 | 0.00 | 0.81 |
| Dissociation (Dissociation) | 26700 | 0.86 | 0.97 | 1.00 | 0.18 | 0.01 | 0.78 |
| DT_anhedonia (Detachment: Anhedonia) | 27124 | 1.23 | 1.23 | 0.68 | -0.80 | 0.01 | 0.78 |
| DT_intimcy_avoid (Detachment: Intimacy avoidance) | 27165 | 0.98 | 1.02 | 0.95 | -0.01 | 0.01 | 0.77 |
| DT_restr_affectivity (Detachment: Restricted affectivity) | 27158 | 1.61 | 1.19 | 0.24 | -1.10 | 0.01 | 0.86 |
| DT_suspiciousness (Detachment: Suspiciousness) | 26661 | 1.39 | 1.37 | 0.47 | -1.17 | 0.01 | NA |
| DT_withdrawal (Detachment: Withdrawal) | 27115 | 1.47 | 1.16 | 0.33 | -1.01 | 0.01 | 0.77 |
| EATA_body_selfest (Anorectic eating disorder: Body weight or shape based self-worth) | 22876 | 1.90 | 1.50 | -0.01 | -1.52 | 0.01 | NA |
| EATA_count_calories (Anorectic eating disorder: Counting calories) | 15375 | 0.31 | 0.91 | 2.99 | 7.84 | 0.01 | NA |
| EATA_exces_exercise (Anorectic eating disorder: Exessive exercise) | 18098 | 0.26 | 0.83 | 3.30 | 10.04 | 0.01 | NA |
| EATA_fear_weightg (Anorectic eating disorder: Fear of weight gain) | 22959 | 1.93 | 1.54 | -0.03 | -1.56 | 0.01 | NA |
| EATA_fear_weightl (Anorectic eating disorder: Fear of weight loss) | 22960 | 0.49 | 1.03 | 2.16 | 3.51 | 0.01 | NA |
| EATA_omit_meal (Anorectic eating disorder: Omitting meals) | 15810 | 0.44 | 0.93 | 2.24 | 4.28 | 0.01 | 0.63 |
| EATA_purging_behv (Anorectic eating disorder: Purging behavior) | 20042 | 0.21 | 0.56 | 3.43 | 13.35 | 0.00 | 0.62 |
| EATA_restricted (Anorectic eating disorder: Restricted eating) | 16965 | 0.86 | 1.26 | 1.21 | 0.12 | 0.01 | NA |
| EATA_time_frames (Anorectic eating disorder: Specific time frames for meals) | 18352 | 0.41 | 0.91 | 2.43 | 5.17 | 0.01 | 0.67 |
| EATB_cope_negaff (Bulimic eating disorder: Eating to cope with negative affect) | 17825 | 1.08 | 1.52 | 0.91 | -0.85 | 0.01 | NA |
| EATB_intrus_thoughts (Bulimic eating disorder: Intrusive thoughts on eating) | 17834 | 0.43 | 1.07 | 2.40 | 4.29 | 0.01 | NA |
| EATB_loss_ctrl (Bulimic eating disorder: Loss of control over eating behavior) | 19532 | 1.22 | 1.47 | 0.74 | -1.02 | 0.01 | 0.66 |
| EATB_overeating (Bulimic eating disorder: Eating notably more than usual) | 17061 | 1.51 | 1.61 | 0.39 | -1.51 | 0.01 | NA |
| EATB_soc_disguised (Bulimic eating disorder: Socially disguised eating behavior) | 23413 | 1.61 | 1.34 | 0.35 | -1.21 | 0.01 | 0.83 |
| EATB_worries (Bulimic eating disorder: Worries around calorie intake and body weight) | 27142 | 2.02 | 1.39 | -0.09 | -1.37 | 0.01 | 0.73 |
| GAD_excess_worry (Generalized anxiety disorder: Excessive worrying) | 27172 | 1.90 | 1.30 | -0.06 | -1.30 | 0.01 | 0.86 |
| GAD_free_float_anx (Generalized anxiety disorder: Free floating anxiety) | 18764 | 1.83 | 1.43 | -0.09 | -1.47 | 0.01 | 0.74 |
| GAD_irritability (Generalized anxiety disorder: Irritability) | 17971 | 1.62 | 1.60 | 0.20 | -1.61 | 0.01 | NA |
| GAD_nervousness (Generalized anxiety disorder: Subjective experience of nervousness) | 18402 | 1.74 | 1.50 | 0.08 | -1.54 | 0.01 | 0.65 |
| Hypochondriasis (Hypochondriasis) | 26864 | 0.90 | 1.24 | 1.16 | 0.01 | 0.01 | NA |
| Manic_sympt (Manic symptoms) | 26560 | 0.38 | 0.59 | 2.26 | 6.30 | 0.00 | 0.60 |
| NA_perseveration (Negative Affectivity: Perseveration) | 27134 | 1.14 | 1.06 | 0.73 | -0.38 | 0.01 | 0.70 |
| NA_sep_insecur (Negative Affectivity: Separation insecurity) | 27170 | 1.82 | 1.22 | 0.09 | -1.16 | 0.01 | 0.82 |
| OCD_sympt (Obsessive compulsive disorder symptoms) | 26547 | 1.05 | 1.01 | 0.95 | 0.11 | 0.01 | 0.76 |
| Panic_attacks (Panic attacks) | 26585 | 1.32 | 1.25 | 0.66 | -0.71 | 0.01 | 0.76 |
| Panic_avoid_behv (Avoidance behavior due to previous panic attacks) | 26253 | 0.91 | 1.39 | 1.13 | -0.34 | 0.01 | NA |
| Panic_doctor_visits (Doctor visits due to previous panic attacks) | 10753 | 0.21 | 0.74 | 3.67 | 12.78 | 0.01 | NA |
| Panic_dysf_thought (Dysfunctional thoughts during panic attacks) | 19502 | 1.00 | 1.23 | 0.96 | -0.31 | 0.01 | 0.84 |
| Panic_fear_recurr (Fear of recurring panic attacks) | 18932 | 1.02 | 1.47 | 0.95 | -0.74 | 0.01 | NA |
| Panic_phys_symp (Physical symptoms during panic attacks) | 18897 | 1.45 | 1.59 | 0.40 | -1.50 | 0.01 | NA |
| PF_affect_comm (Personality functioning: Affect communication) | 27116 | 1.34 | 1.21 | 0.51 | -0.97 | 0.01 | 0.80 |
| PF_affect_diff (Personality functioning: Affect differentiation) | 27152 | 1.68 | 1.29 | 0.19 | -1.27 | 0.01 | 0.76 |
| PF_affect_tolrnce (Personality functioning: Affect tolerance) | 27138 | 1.55 | 1.24 | 0.33 | -1.09 | 0.01 | 0.72 |
| PF_anticipation (Personality functioning: Anticipating behavior of others) | 26165 | 1.74 | 1.26 | 0.04 | -1.20 | 0.01 | NA |
| PF_forming_relshps (Personality functioning: Forming relationships) | 27152 | 1.60 | 1.32 | 0.27 | -1.26 | 0.01 | 0.82 |
| PF_holistic_percptn (Personality functioning: Holistic perception of others) | 27063 | 1.34 | 1.09 | 0.51 | -0.63 | 0.01 | 0.65 |
| PF_identity (Personality functioning: Identity problems) | 27172 | 1.23 | 1.08 | 0.69 | -0.51 | 0.01 | 0.85 |
| PF_impulse_reg (Personality functioning: Impulse regulation) | 27141 | 1.05 | 1.09 | 0.91 | -0.18 | 0.01 | 0.85 |
| PF_intern_relshp_mod (Personality functioning: Internal model of relationships) | 27127 | 1.58 | 1.18 | 0.28 | -1.08 | 0.01 | 0.89 |
| PF_self_est_reg (Personality functioning: Regulation of self-esteem) | 27168 | 1.97 | 1.07 | -0.07 | -0.91 | 0.01 | 0.70 |
| PF_self_reflection (Personality functioning: Self reflection) | 27139 | 1.50 | 1.29 | 0.36 | -1.19 | 0.01 | 0.77 |
| PS_eccentricity (Psychoticism: Eccentricity) | 27062 | 1.25 | 1.21 | 0.62 | -0.82 | 0.01 | 0.85 |
| PS_percept_dysreg (Psychoticism: Perceptual dysregulation) | 26863 | 0.48 | 0.89 | 2.07 | 3.74 | 0.01 | 0.87 |
| PS_unusual_beliefs (Psychoticism: Unusual beliefs) | 27062 | 0.47 | 0.76 | 2.01 | 4.10 | 0.00 | 0.82 |
| Psychotic_symptoms (Psychotic symptoms) | 27148 | 0.57 | 0.81 | 1.79 | 3.00 | 0.00 | 0.78 |
| SAD_attractive_pers (Social anxiety when interacting with attractive persons) | 19460 | 1.14 | 1.49 | 0.79 | -0.98 | 0.01 | NA |
| SAD_avoidance (Social anxiety: avoidance of interactions with strangers) | 15215 | 1.18 | 1.50 | 0.70 | -1.13 | 0.01 | NA |
| SAD_being_observ (Social anxiety with doing tasks while being observed) | 19469 | 0.87 | 1.37 | 1.17 | -0.19 | 0.01 | NA |
| SAD_devaluation (Social anxiety: Fear of devaluation) | 15588 | 1.63 | 1.61 | 0.22 | -1.63 | 0.01 | 0.67 |
| SAD_eating (Social anxiety when eating in front of others) | 15254 | 0.69 | 1.27 | 1.52 | 0.80 | 0.01 | NA |
| SAD_performing (Social anxiety when performing in front of others) | 19910 | 1.04 | 1.21 | 0.87 | -0.48 | 0.01 | 0.78 |
| SAD_soc_inter (Social anxiety in social interactions) | 27168 | 1.51 | 1.48 | 0.36 | -1.40 | 0.01 | 0.88 |
| Somatic_symptoms (Somatic symptoms) | 27173 | 1.04 | 0.93 | 0.95 | 0.20 | 0.01 | 0.86 |
| Spec_phob_animals (Specific phobia: Animals) | 20234 | 0.89 | 1.28 | 1.12 | -0.10 | 0.01 | NA |
| Spec_phob_avoid (Specific phobia: Avoidance of feared objects) | 20233 | 1.36 | 1.53 | 0.47 | -1.40 | 0.01 | NA |
| Spec_phob_blood (Specific phobia: Blood-injury phobia) | 20263 | 0.37 | 0.99 | 2.57 | 5.32 | 0.01 | NA |
| Spec_phob_fear (Specific phobia: Excessive fear of certain objects) | 27171 | 1.18 | 1.28 | 0.72 | -0.80 | 0.01 | 0.89 |
| Subst_use_alc (Substance use: Regular use of alcohol) | 27155 | 0.34 | 0.72 | 2.53 | 6.18 | 0.00 | 0.94 |
| Subst_use_drug (Substance use: Regular use of drugs) | 27075 | 0.25 | 0.62 | 3.16 | 10.66 | 0.00 | 0.97 |
