# Supplementary material for: Examining the role of personality functioning in a hierarchical taxonomy of psychopathology using two years of ambulatory assessed data
Source: Transl Psychiatry. 2024 Aug 24;14:340. doi: 10.1038/s41398-024-03046-z (PMC11344763; doi:10.1038/s41398-024-03046-z)
Supplement: Supplementary file 3 — Supplementary Material 3 [file 41398_2024_3046_MOESM3_ESM.docx]

### Supplemental material 3: Bass-ackward-analysis

### Agglomerative Cluster Analysis based on 98 scales using Ward’s Hierarchical method
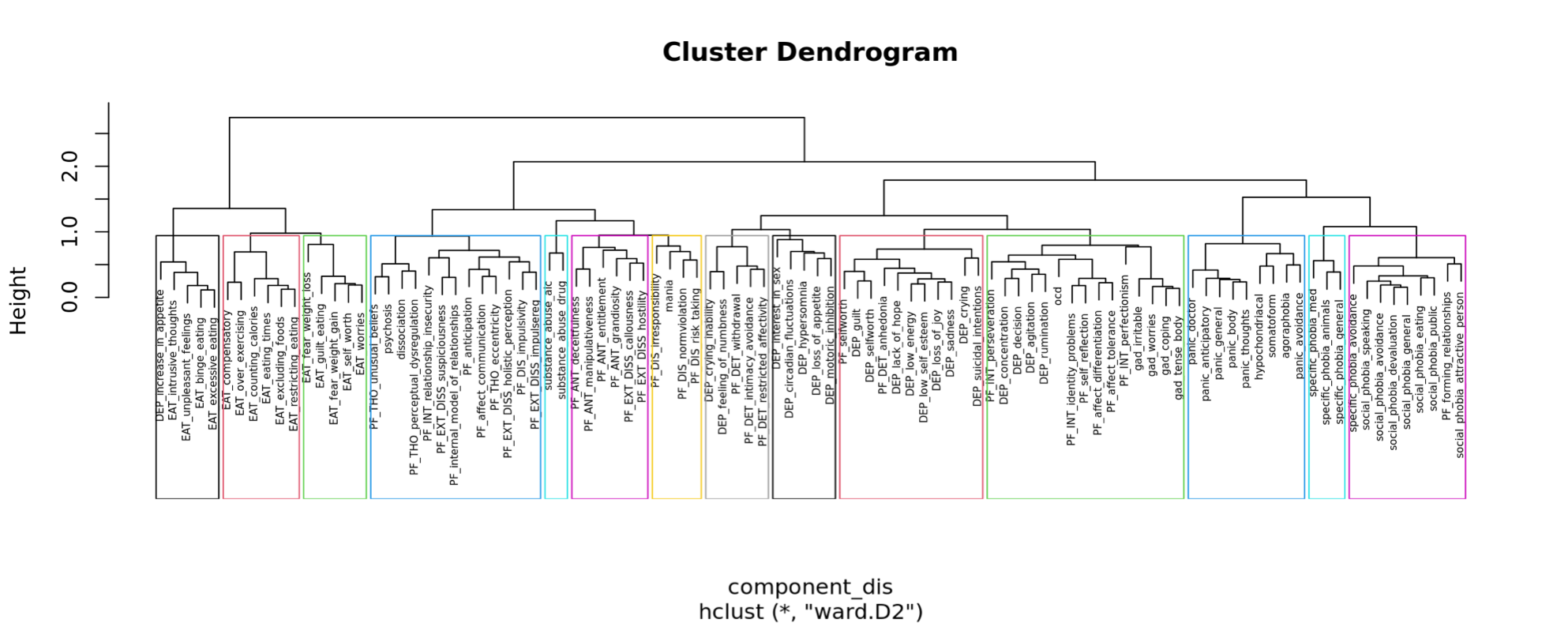


### Redundant and artefactual components identified in the bass-ackwards procedure

Bass-ackwards procedure according to (1) extracts an increasing number of components (1, 2, 3, ..., n) in a top-down process and calculates the correlations between the components of subsequent hierarchical levels (2). The top level component in these models is often called “p-factor”. Based on the correlations between all levels and components, a hierarchical structure of psychopathology best fitting the data is identified involving three steps: First, identifying and removing redundant components with *r* > .9 and a congruence coefficient for loadings > .95, then identifying and removing artefactual components based on the examination of the cluster analytic hierarchy of the data (using Ward’s agglomerative clustering method), and adding correlations from lower- to higher-order components that are not accounted for hierarchically in a third step.

Redundancy (i.e., *r* > .90 and congruence coefficient > .95) of components at the bottom level with components from higher levels are depicted in light green. Here, according to the procedure described in [Forbes (2023)](https://www.zotero.org/google-docs/?YmEfxs), only the bottom component remains because of greater specificity of the constructs in the model. Redundancy of higher level components with components from lower levels that do not include the bottom layer are depicted in dark green. Here, only the component highest to the top of the hierarchy remains, that is, the broadest manifestation subsuming lower level constructs. Components close to redundancy were N1 and J2 (*r* = .89), N1 and E2 (*r* = .84), N3 and E5 (*r* = .83), N13 and L10 (*r* = .87), N5 and F5 (*r* = .88), N7 and H6 (*r* = .91), J8 and H7 (*r* = .89), and N14 and I9 (*r* = .82).

After removing the redundant components in the hierarchical structure, three components could be identified as artefactual and being absent in the agglomerative cluster analysis (see Figure with cluster dendogram above): K8 with loadings on depression (N3) and eating disorder thoughts (N13) emerging from J3 (mainly generalized anxiety [GAD] and obsessive compulsive disorder [OCD]), J9 with positive loadings on specific phobia (N12) and negative loadings on D´detachment (N10), emerging from I6 (mainly thought disorder) and C2 with positive loadings on panic (N3), thought (N7), antagonism (N11) and disinhibition (N14), emerging from B1 (internalizing).

The third step in the identification of the hierarchical structure was to include correlations larger than .3 (3) of the lower-level components with the remaining higher-order components that were not already accounted for hierarchically. PF problems (N8) showed significant cross-loadings with G3 (GAD and OCD), detachment (N10) with G1 (somatic depression symptoms). The hierarchically unrelated components eating disorder related thoughts (N13) and specific phobia (N12) showed their highest correlations with higher-order eating disorder (B2) and higher-order fear component (D2), respectively.

References

1. Forbes MK. Improving hierarchical models of individual differences: An extension of‬
